# Biofluid‐based biomarkers to predict onset of Alzheimer's disease neuropathology

**DOI:** 10.1002/alz70856_096587

**Published:** 2025-12-24

**Authors:** Henrik Zetterberg, Douglas R. Galasko

**Affiliations:** ^1^ Department of Psychiatry and Neurochemistry, Institute of Neuroscience and Physiology, the Sahlgrenska Academy, University of Gothenburg, Molndal, Sweden; ^2^ Wisconsin Alzheimer's Disease Research Center, University of Wisconsin‐Madison, School of Medicine and Public Health, Madison, WI, USA; ^3^ University College London, London, United Kingdom, London, United Kingdom; ^4^ UCSD, San Diego, CA, USA

## Abstract

Biofluid‐based biomarkers detect amyloid, tau and neurodegeneration (ATN) pathophysiological changes prior to onset of symptoms. But are there biomarkers that can predict the onset of these pathologies, i.e., predict AD before the brain changes have developed? Genetics, proteomics and ultrasensitive immunoassays were performed in 193 subjects with AD across the cognitive spectrum. In *APOE* ε4 carriers, i.e., in individuals at increased risk of AD, with normal amyloid β and cognition, CSF biomarkers for neuronal injury were normal. Compared to the control group, ten proteins (apoE4 fragment, tau, BACE1, β‐nerve growth factor [NGF], macrophage inflammatory protein‐1β [CCL4], osteopontin [SPP1], AXL receptor tyrosine kinase [AXL], heparin‐binding EGF‐like growth factor [HBGF], carbonic anhydrase‐1 [CA1], interferon gamma‐induced protein‐10 [CXCL10]) showed altered levels. The top KEGG pathway enriched was the Toll‐like receptor signaling pathway (pFDR = 0.00033; including SPP1, CXCL10, and CCL4). Upregulated amyloidogenic APP‐processing and increased neuronal and astroglial activity may precede amyloid positivity according to CSF biomarkers. Data replicating these findings in larger cohorts will be presented.

